# Computing structure-based lipid accessibility of membrane proteins with mp_lipid_acc in RosettaMP

**DOI:** 10.1186/s12859-017-1541-z

**Published:** 2017-02-20

**Authors:** Julia Koehler Leman, Sergey Lyskov, Richard Bonneau

**Affiliations:** 1 Center for Computational Biology, Flatiron Institute, 162 Fifth Avenue, New York, NY 10010 USA; 2 Departments of Biology and Computer Science, Center for Genomics and Systems Biology, New York University, New York, NY 10003 USA; 3 Department of Chemical and Biomolecular Engineering, Johns Hopkins University, Baltimore, MD 21218 USA

**Keywords:** Membrane proteins, Structure prediction, Lipid accessibility, Accessible surface area, Rosetta software

## Abstract

**Background:**

Membrane proteins are underrepresented in structural databases, which has led to a lack of computational tools and the corresponding inappropriate use of tools designed for soluble proteins. For membrane proteins, lipid accessibility is an essential property. Although programs are available for sequence-based prediction of lipid accessibility and structure-based identification of solvent-accessible surface area, the latter does not distinguish between water accessible and lipid accessible residues in membrane proteins.

**Results:**

Here we present mp_lipid_acc, the first method to identify lipid accessible residues from the protein structure, implemented in the RosettaMP framework and available as a webserver. Our method uses protein structures transformed in membrane coordinates, for instance from PDBTM or OPM databases, and a defined membrane thickness to classify lipid accessibility of residues. mp_lipid_acc is applicable to both α-helical and β-barrel membrane proteins of diverse architectures with or without water-filled pores and uses a concave hull algorithm for surface-residue classification. We further provide a manually curated benchmark dataset that can be used for further method development.

**Conclusions:**

We present a novel tool to classify lipid accessibility from the protein structure, which is applicable to proteins of diverse architectures and achieves prediction accuracies of 90% on a manually curated database. mp_lipid_acc is part of the Rosetta software suite, available at www.rosettacommons.org. The webserver is available at http://rosie.graylab.jhu.edu/mp_lipid_acc/submit and the benchmark dataset is available at http://tinyurl.com/mp-lipid-acc-dataset.

**Electronic supplementary material:**

The online version of this article (doi:10.1186/s12859-017-1541-z) contains supplementary material, which is available to authorized users.

## Background

Membrane proteins carry out a variety of essential functions and are targeted by over half of drugs in use [1], yet only make up ~2% of proteins in the Protein Data Bank due to difficulties in structure elucidation. The dearth of structures has in turn led to a lack of prediction tools, which have typically focused on either α-helical or β-barrel membrane proteins [2], or on specific features like the prediction of membrane pores [3]. One important characteristic of membrane proteins is accessibility to the lipid environment of the bilayer. Knowledge of lipid accessibility is important for our understanding of membrane protein structure, stability [4], interactions inside the bilayer, the development of membrane protein energy functions, the development of sequence-based predictors, and as an indicator of binding interfaces. While sequence motifs for helix-helix interactions in the membrane have been well-studied [5], the broader picture of large protein-protein interfaces in the membrane is still developing.

Prediction of lipid accessibility is not trivial: the protein ‘interior’ can either be water accessible, in the case of pores, or buried hydrophobic residues. For the latter, the hydrophobicity profile of buried residues is similar to lipid-facing residues, hence hydrophobicity as a solitary feature would be insufficient for classification. Solvent accessible surface area (SASA) is useful to identify accessibility of the residue to the solvent, yet it does not distinguish between lipid or water accessibility. Further, geometric considerations might be useful for the structures of β-barrels, as sidechains typically either face into the barrel or away from it, but this distinction is typically less clear for α-helical proteins. Therefore, a combination of different features is required for accurate prediction of lipid accessible residues.

The problem is further complicated by the fact that accessibility to lipid relies on protein embedding in the membrane and experimental measurements of membrane embedding features (like depth, angle and membrane thickness) are challenging. Lipid molecules are only present in a few crystal structures, and the model membrane used for crystallization or structure determination is very different from a native membrane environment because it lacks the exact lipid composition [6], membrane thickness, asymmetry [7], lateral pressure [8] and shape [9] (compare micelles, bicelles, nanodiscs, lipidic cubic phases vs. a flat or curved bilayer). Protein embedding in the membrane is therefore predicted using computational approaches that rely on different score functions, such as TMDET [10], PPM [11], and iMembrane [12]. However, benchmarking of these tools is difficult without high-resolution experimental data. Similarly, for lipid accessibility there are no compiled structural databases to test the performance of a predictor from the protein structure, hence expert manual curation of such a database is required.

A range of **sequence-based predictors** is available that specialize in transmembrane span or topology prediction for α-helical bundles or β-barrels, SASA or lipid accessibility. Transmembrane span predictors classify residues as *in the membrane* vs. *in solution* and are able to achieve prediction accuracies >90% in the two-state scenario [13], [14]. Topology predictors [15], [16] classify residues as *inside/outside* the cell or organelle and *in the membrane* (for definitions for the OPM (Orientations of Proteins in Membranes) database [11], see http://opm.phar.umich.edu/about.php?subject=topology). SASA predictors classify residues as *buried* or *exposed* to the solvent without specifying whether the solvent is water or lipid. They typically use machine learning (for example, support vector machine) approaches and report prediction accuracies in the range of 70-75%: the ASAP tool can be used for both α-helical and β-barrel membrane proteins [17], the MPRAP method is optimized for SASA prediction of both soluble and transmembrane regions of membrane proteins [18], and the TMExpo tool predicts embedding angles in addition to SASA [19]. Further, pore-lining residues and channels can be predicted from the protein sequence using the recently developed tool PRIMSIPLR [3], which achieves a prediction accuracy around 86%. Lipid accessibility (*lipid exposed* versus *lipid buried*) can be predicted from the protein sequence with the LIPS server [20], which uses contact maps between helical faces and residue conservation for classification. It achieves a prediction accuracy of 88%, according to the authors.

**SASA prediction directly from protein structure** can be obtained with a few early predictors (Naccess [21], MSMS [22], GetArea: http://curie.utmb.edu/getarea.html [23]); unfortunately, some of these methods lack benchmarking results, were only tested on a few low-resolution crystal structures or lack availability as a web server and/or lack documentation. The recently developed, well-documented 3V web interface for volume, solvent exclusion and channel prediction does not compute SASA values per residue [24].

Here, we present a method to identify lipid accessible residues from a protein structure that is already transformed into membrane coordinates. Our method uses a 2D concave hull algorithm on a point cloud, which is generated by projecting Cα coordinates from horizontal slices of the protein within the bilayer, onto the plane of the membrane. For each slice, the convex hull, the concave hull and a ‘concave shell’ are computed, through which lipid accessible residues are identified (see Implementation and Fig. 2). The classification of the concave shell is output as adjusted B-factors in the PDB file, which can be easily visualized or extracted for further analysis. We compute prediction accuracies on a manually curated benchmark dataset available for download at http://tinyurl.com/mp-lipid-acc-dataset. Our method mp_lipid_acc is also publicly available as a webserver on ROSIE [25] at http://rosie.graylab.jhu.edu/mp_lipid_acc/submit. To our knowledge, this is the first tool to classify lipid exposure of residues from a protein structure. Our method is applicable to both α-helical and β-barrel membrane proteins of any architecture and will be useful for a variety of problems from score function derivation, development of sequence-based predictors and structural modeling.

## Implementation

### Curation of training and benchmark datasets

We created a small dataset of 14 proteins for the development of the algorithm (Additional file 1: Table S1) and to find an optimal parameter set. These proteins cover a wide range of protein folds from one or two transmembrane helices, helical bundles with and without smaller pores (for instance GPCRs, aquaporins), small and large helical pores, transporters (ABC transporter), ion channels with voltage sensor domains, oblong helical channels (for instance chloride channel), β-barrels with and without internal domains, β-barrel monomers and trimers and cigar shaped β-barrels and pore-forming toxins. The PDBIDs for this dataset were 1afo, 1ek9, 1fep, 1kpk, 1qd6, 2kix, 2r9r, 2rh1, 2wcd, 3emn, 3ne2, 3wmf, 4tnw, 7ahl.

We curated a benchmark dataset for testing our method (Additional file 1: Table S2): all membrane protein chains were downloaded from the PDBTM database [26], which were then culled with the PISCES server [27] with the following parameters: sequence identity ≤ 25%, resolution ≤ 3.0 Å, R-factor cutoff 0.3, sequence length 40–10,000, include non-Xray entries, exclude Cα-only entries, cull by chain. We then excluded all EM structures with resolutions > 3 Å and removed all XFEL structures with resolution “0.0” or proteins for which no method was specified. Further, we removed photosynthetic reaction centers and photosystems I and II as they have very loosely packed helices complexed with a large number of interstitial chlorophyll molecules. We also removed the proteins present in the training dataset.

The structures in each set were downloaded from the PDBTM database [26], for which the proteins are already transformed into the membrane coordinate frame: the membrane center is defined as the origin at (x, y, z) = (0, 0, 0) and the membrane normal vector lies along the z-axis with (0, 0, 1). To compute the membrane embedding of a protein structure, PDBTM uses the TMDET algorithm [10, 28] that fits the membrane embedding according to an objective function that contains measures of hydrophobicity and structural information regarding the Cα-trace within a protein slice, such as straightness, turns and termini. Initially, we downloaded structures from the OPM database [11], but through visual inspection of the entire database we realized that the protein embedding seems generally better in PDBTM. The structures were then cleaned from additional atoms (such as ligands and co-factors), renumbered and the span files were computed as described previously [29] with a fixed membrane thickness of 30 Å. We used a fixed membrane thickness to avoid a potentially circular influence of the thickness prediction from PDBTM onto our benchmark dataset.

We ran our algorithm over the benchmark dataset (204 proteins), which outputs the lipid accessibility as a modified B-factor column in the PDB file. We then manually corrected classification errors by visualizing each protein in PyMOL [30], coloring it according to B-factors, and then manually adjusting the B-factors for each incorrectly classified residue. Our benchmark dataset is described in Additional file 1: Table S2 and is available for download at http://tinyurl.com/mp-lipid-acc-dataset.

### Algorithm overview

**Fig. 1:**
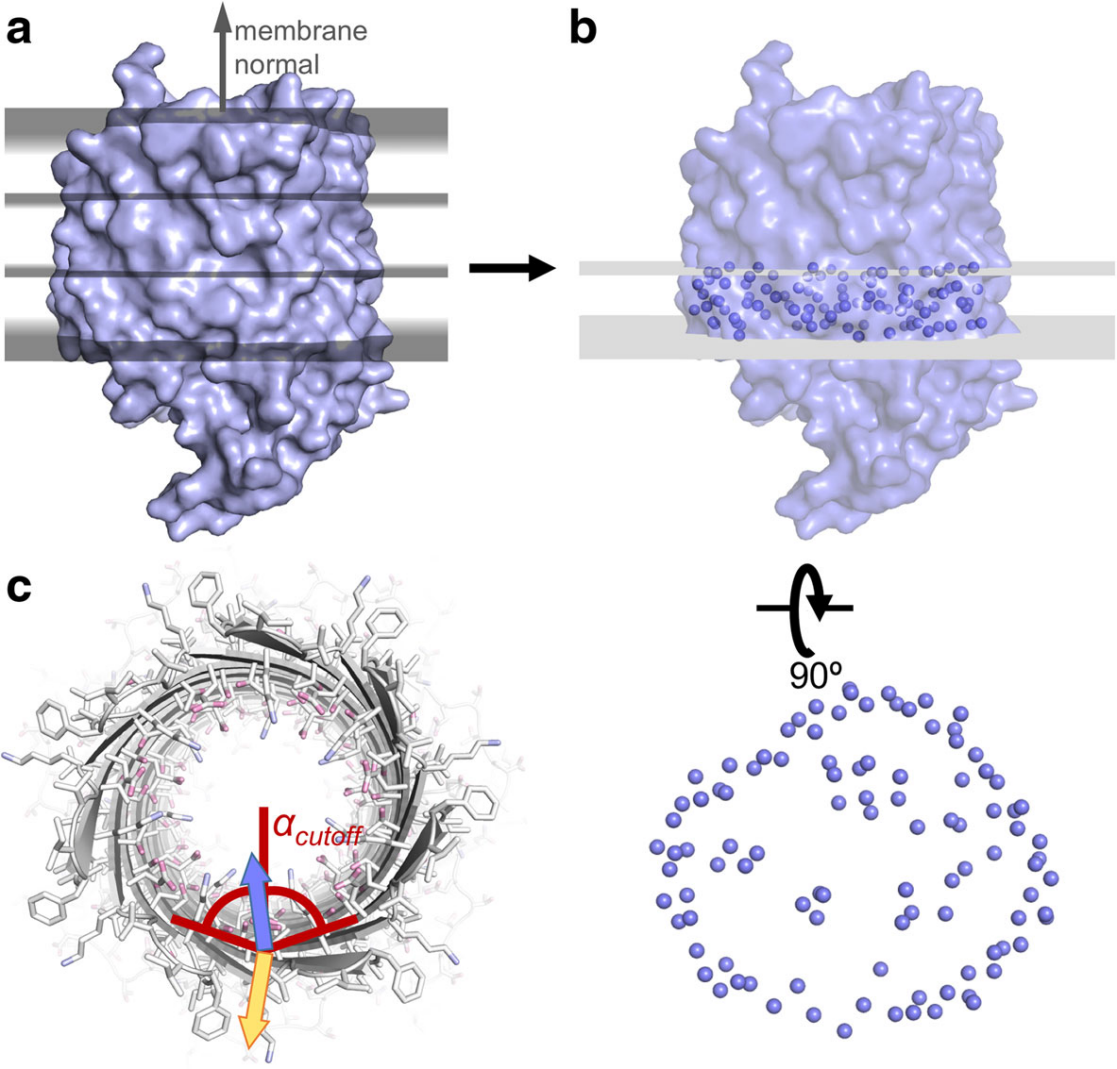
Algorithm. In our algorithm mp_lipid_acc, the protein is cut into horizontal slices (**a**). In each slice, the coordinates of the Cα atoms are projected onto the xy-plane, from which points first the convex hull, then the concave hull and then the ‘concave shell’ are computed ((**b**) and see implementation). For β-barrels, the center-of-mass in each slice is computed from the coordinates of the Cα atoms and only residues which are part of the concave shell and have a Cα-Cβ-COM angle larger than a cutoff value of 65° are classified as lipid accessible (**c**). For instance, the residue with a sidechain orientation represented by the *yellow* vector in (**c**) would be lipid accessible, whereas the sidechain in *blue* would be lipid inaccessible

**Fig. 2:**
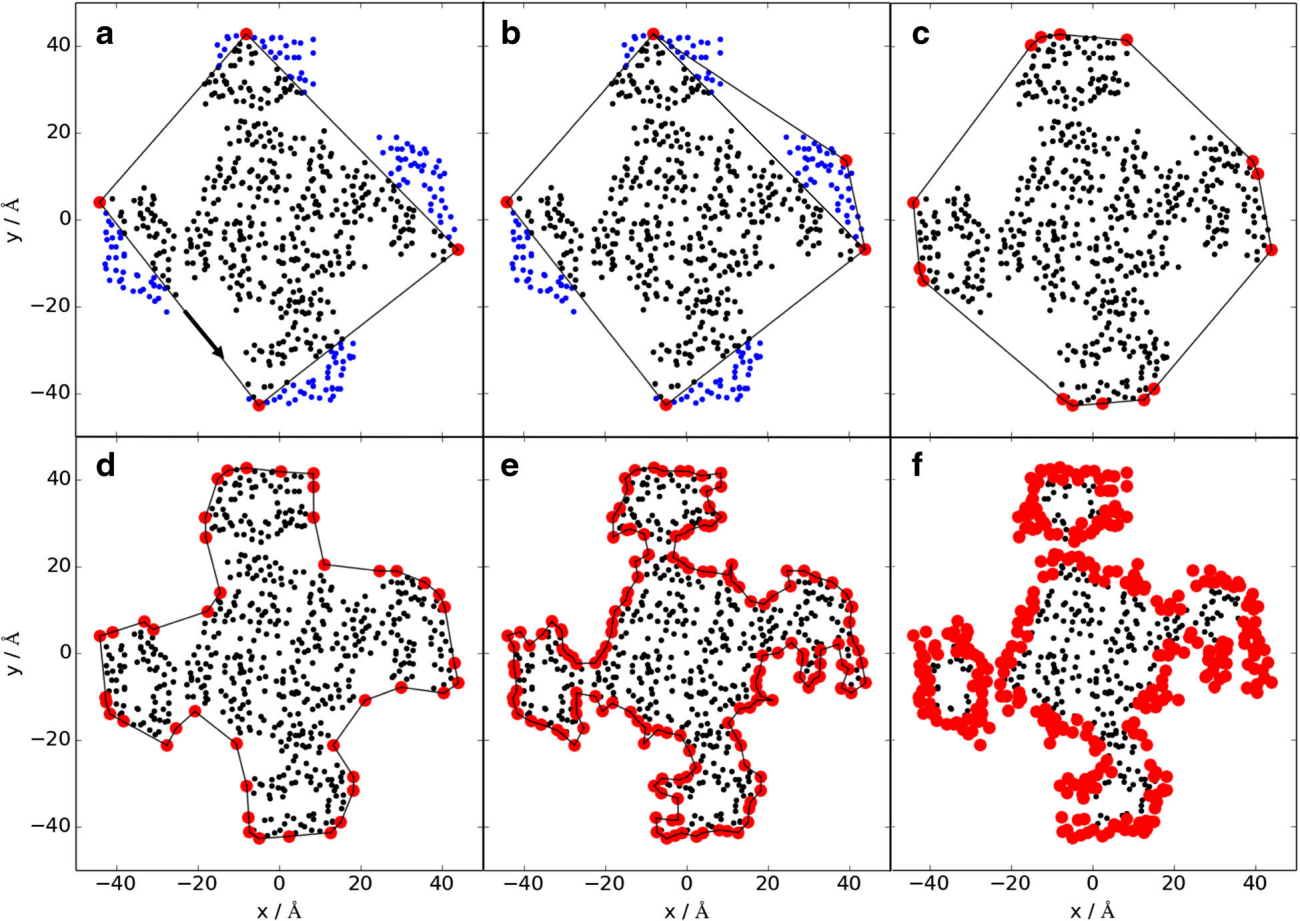
Convex and concave hull algorithms. Illustration of the convex hull, concave hull and ‘concave shell’ from a 2D point cloud that was projected onto the xy plane from the 3D structure. The example protein is the sodium channel with PDBID 4DXW and the points in *blue* are outside the hull, the points in *black* are inside the hull and the points in *red* are part of the hull. The convex hull algorithm starts by connecting the points with smallest and largest x and y values in counter-clockwise direction and identifying the points inside the hull (**a**). The hull is extended to a convex hull by connecting two points on the boundary, finding the point in the outside list that is farthest away from the two boundary points and that is in clockwise direction, and adding this point to the boundary (**b**). The points within the triangle between the old two boundary points and the added one are then classified as inside the hull. These steps are repeated until all points are inside the convex hull (**c**). The concave hull is then computed by finding the longest distance between two connected points on the boundary, finding the point inside the hull that has the smallest enclosing angles with respect to the two original boundary points, and adding this new point to the hull boundary. The distance cutoff defines the ‘resolution’ of the hull with a distance cutoff of 15 Å in (**d**) and 5 Å in (**e**). This process is repeated until all distances between connecting points are smaller than the cutoff distance. Lastly, a concave shell is computed by including points within an xy distance radius from the original boundary points (from **e** to **f**). All boundary points in F are now part of the concave shell and classified as lipid exposed

To identify lipid exposed residues on the outer perimeter of the protein, we approximate the membrane protein as a stack of 2D slices and then within each slice, use a 2D concave hull algorithm to determine water and lipid accessible boundaries. For this, the protein was cut into 10 Å thick horizontal slices along the membrane normal (Fig. 1a). For each slice, the Cα atoms were projected onto the xy plane (Fig. 1b), from which points the convex hull was computed (see below and Fig. 2). The convex hull is the smallest set of points on the outer perimeter of the 2D point cloud that encloses the entire point cloud; it is computed by a *QuickHull* algorithm [31, 32] (see below). From the convex hull (Fig. 2c), we computed the concave hull [33] (Fig. 2d/e), which defines the set of points on the outer perimeter of the point cloud and encloses the point cloud within each slice by potentially concave surfaces (see below). We then computed the concave shell that includes points (Cα atoms) whose xy coordinates are within a radius of the original boundary points (Fig. 2f). Concave shells were computed for each stacked horizontal slice in the membrane region.

Residues with Cα atoms in the concave shell are classified as lipid exposed with the following exceptions: (1) if the number of TM spans is smaller or equal 2, all residues in the membrane region are classified as lipid exposed; (2) for β-barrels, we make the distinction of whether the sidechain faces into the interior of the barrel or not. For each slice, we therefore compute the center-of-mass (COM) of the Cα atoms and only classify residues with the Cβ-Cα-COM angle larger than 65° as lipid accessible (for Glycine, we used 2HA to represent ‘Cβ’ – Fig. 1c). (3) For smaller helical bundles with 7 or less TM spans, the concave shell algorithm over-predicted lipid accessible residues in the interior of the protein. To counter balance this over-prediction, we took into account an angle cutoff of 45° as described above.

### Convex and concave hulls

A **convex hull** is a subset of points in a point cloud that engulfs all points in the cloud via convex surfaces (Fig. 2c). The convex hull algorithm starts with a classification of a 2D point cloud into three lists: inside the hull (black points in Fig. 2a), outside the hull (blue points in Fig. 2a), and part of the hull (i.e. on the boundary – red points in Fig. 2a). The *Quickhull* algorithm starts by classifying all points as outside the hull [31], [32]. Then, the points with the smallest x value, smallest y, largest x and largest y are connected and moved to the list of points on the boundary. The points inside the rectangle are moved to the inside list (Fig. 2a). Then, two neighboring points in the boundary list are connected by a line and the point outside with the largest distance in clockwise direction is moved to the boundary (Fig. 2b), while the points inside this triangle are added to the point list inside the hull. This last step is repeated until all points are either inside the hull or on the boundary and the outside list is empty. The convex hull algorithm identifies the points on the hull that make up a convex shape (Fig. 2c). However, it fails to identify points on the hull that encompass the smallest surface area.

The **concave hull** on the other hand (Fig. 2d), engulfs all points with the smallest surface area, which requires ‘cutting into’ the boundaries of the convex hull to create concave surfaces. Starting from the convex hull, two neighboring points on the boundary are connected by a line [33]. If the distance of the line segment is larger than a pre-defined distance cutoff (see Fig. 2d), the point inside the hull (counter-clockwise) is identified that has the smallest sum of angles to the two original boundary points. This point is added to the boundary and this last step is repeated until all the distances between neighboring points in the boundary list are smaller than the pre-defined cutoff. The distance cutoff is required because there is no unique solution to the classification problem as to which points are part of the concave hull. The distance cutoff is therefore a measure similar to a ‘resolution’ that defines how rugged the concave surfaces are (compare Fig. 2d and e).

To extend the information of the concave hull back into three dimensions, we classified points (i.e. Cα atoms) within a certain xy-distance (‘shell radius’) from the points on the boundary as part of the boundary—we call this the **concave shell** (moving from Fig. 2e to f).

### Adjustable parameters in the algorithm

Optionally adjustable parameters for this application are: (1) the **slice width** (Fig. 1a) as the width of the horizontal slices for which the concave shells are computed. As only Cα atoms are considered and to avoid overfitting of the convex shell due to data sparsity, the default slice width is set to 10 Å, corresponding to approximately 1/3 of the thickness of a physical membrane bilayer. The three slices therefore extend over the inner and outer leaflets and the space in between. Further, the membrane thickness should be an integer multiple of the slice width to avoid data sparsity in the last slice. The current membrane thickness is fixed at 30 Å. (2) **Distance cutoff** (compare Fig. 2d and e): The ‘resolution’ of the concave hull is defined by the distance cutoff between points on the hull boundary. 2D line segments between neighboring points on the hull are ‘cut in’ if their distance is longer than the distance cutoff. The default value for the distance cutoff is 10 Å, which is approximately the distance between two Cα atoms on the same side of neighboring helices. (3) **Shell radius** (Fig. 2e to f): To map the 2D concave hull back into three dimensions, Cα atoms with xy coordinates within a certain radius (shell radius) of the original boundary points are added to the hull, which we call the concave shell. The default shell radius is 6 Å, which is about half the diameter of an α-helix. (4) **Angle cutoff** (Fig. 1c): To distinguish between sidechains facing water-filled interiors in β-barrels from sidechains facing the lipid environment, we defined a cutoff for the Cβ-Cα-COM angle. The default value for the angle cutoff is 65°, which is empirically determined and smaller than 90° to account for the curvature of β-barrels. (5) To classify lipid accessibility we distinguish between an α-helical bundle (for which we don’t use an angle cutoff) or β-barrel membrane protein (for which we use an angle cutoff), which refers to the secondary structure facing the lipid (i.e. barrel interior secondary structure is not considered). The type is auto-detected based on the prevalent secondary structure in the membrane, as computed by DSSP [34]. In the rare scenario that auto-detection fails, the secondary structure type can be set by the user.

If a residue is classified as lipid accessible, its B-factor is set to 50, otherwise it is set to 0. The PDB structure with the adjusted B-factor is output and can be visualized in PyMOL (The PyMOL Molecular Graphics System, Version 1.8 Schrödinger, LLC) with the provided script color_b-factor.pml.

## Results and Discussion

We present an algorithm that classifies lipid exposed residues in membrane protein structures. It is applicable to monomeric and oligomeric α-helical and β-barrel membrane proteins with and without pores. Our algorithm uses protein structures transformed into membrane coordinates and a fixed membrane thickness and is applicable to a wide range of protein architectures. Classification is achieved through a 2D concave hull algorithm applied to the point cloud of membrane embedded Cα atoms projected onto the membrane plane. mp_lipid_acc then creates a PDB structure file with modified B-factors that can then be visualized by a provided PyMOL script. We further provide a publicly accessible webserver to run the classification, which is implemented in the ROSIE environment [25], accessible at http://rosie.graylab.jhu.edu/mp_lipid_acc/submit. Additionally, we make our manually curated benchmark dataset available to the public (http://tinyurl.com/mp-lipid-acc-dataset), which will be useful for the development of sequence-based predictors and membrane protein scoring functions.

**Fig. 3:**
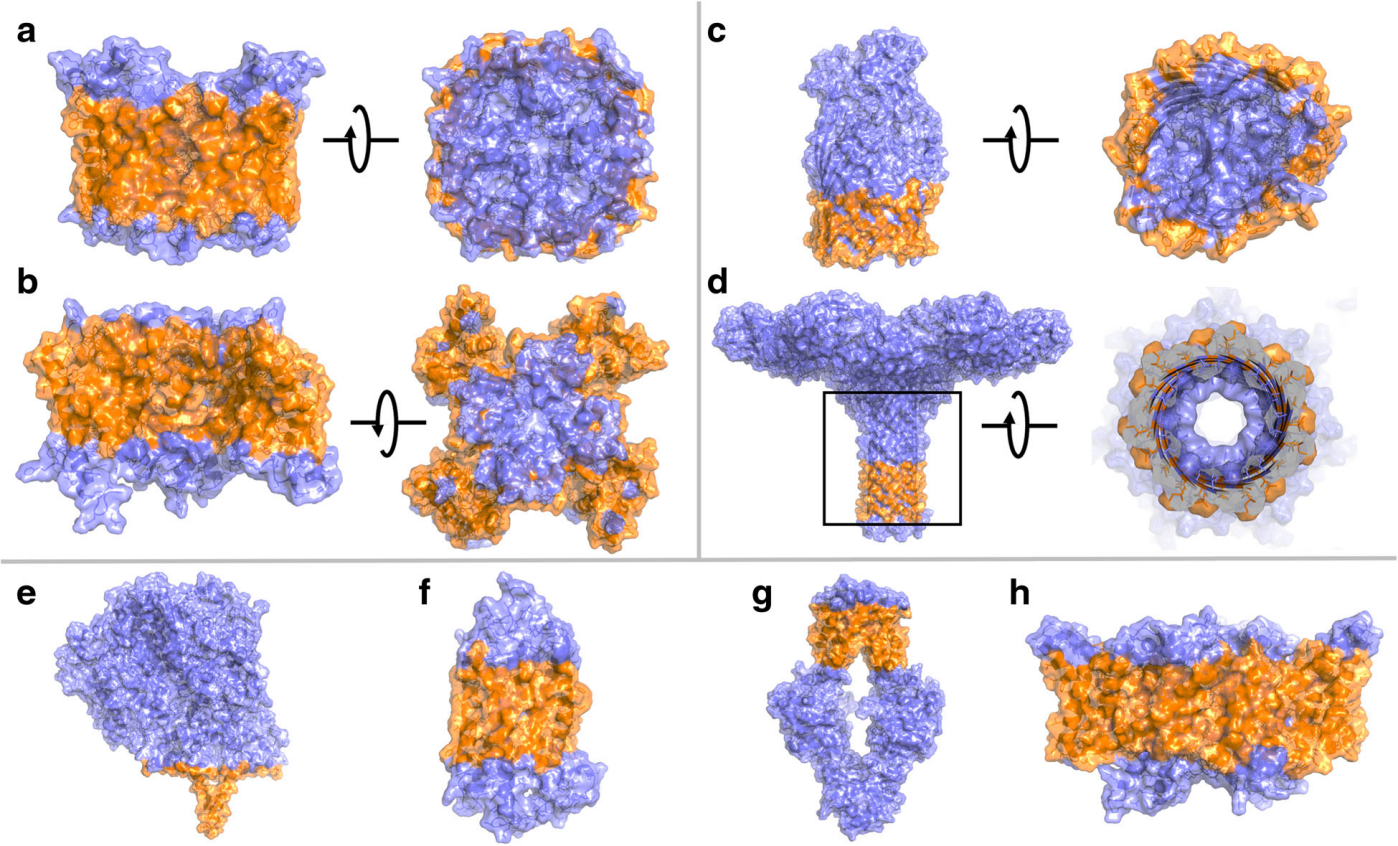
Classifications on selected examples from the benchmark dataset with diverse architectures. Details about the individual proteins are provided in Table 2. In panel **d**, the pore of the protein (*framed*) is shown on the right, where the residues that protrude into solution, are clipped to show the prediction for the pore residues

The prediction accuracies for our benchmark set are shown in Table 1 with counts for true positives, true negatives, false positives and false negatives. Accuracy, sensitivity and specificity are also computed. As a baseline for comparison, we used the prediction of rASA with a cutoff of 0.2. Table 1 shows that while rASA achieves almost as high prediction accuracies as our predictor, it is unable to distinguish between lipid accessible and water accessible residues, as demonstrated by the low sensitivity. In contrast, our simple algorithm correctly identifies lipid exposed residues, achieving a prediction accuracy of 91.2% (Table 1, Fig. 3, Table 2 and Additional file 1: Table S2), with consistently high sensitivity and specificity (~90%).

**Table 1:** Prediction accuracies in percent for the benchmark set. The first row indicates a prediction using relative accessible surface area in the membrane (cutoff 0.2) without lipid accessibility classification. The second row shows results for predicted lipid accessibility with our concave hull algorithm. Note that mp_lipid_acc is able to identify lipid exposed residues, giving rise to an almost 20% increase in sensitivity over a standard rASA algorithm

	TP	TN	FP	FN	acc	sens	spec
rASA	26526	119406	7727	10602	88.8	71.4	93.9
hull	33191	116548	10585	3937	91.2	89.4	91.7

*TP* number of residues predicted as true positives, *TN* true negatives, *FP* false positives, *FN* false negatives, *acc* accuracy = (TP + TN)/(TP + TN + FP + FN); sens = TP/(TP + FN); spec = TN/(TN + FP)

**Table 2:** Proteins for which predictions are shown in Fig. 3. A/B denotes α-helical vs. β-barrel membrane proteins. The complete benchmark dataset is shown in Additional file 1: Table S2

PDBID	A/B	Feature	Family	Subfigure
3D9S	A	aquaporin	aquaporin	A
4DXW	A	ion channel	sodium channel	B
3CSL	B	filled barrel	heme binding	C
3W9T	B	pore-forming toxin	lectin	D
2J7A	A	1-2TM	nitrite reductase	E
1U19	A	GPCR	bovine rhodopsin	F
3TUI	A	ABC transporter	methionine transporter	G
4ENE	A	ClC channel	chloride channel	H

We aimed to classify residues in well-packed α-helical proteins, helical pores, plain β-barrels, β-barrels with plug domains, monomeric and multimeric membrane proteins with and without pores, and multimeric ion channels with a large lipid exposed surface area due to their voltage sensor domains (Fig. 3). Because we are using a concave hull algorithm, mp_lipid_acc is applicable to a wide range of protein architectures that have perimeters of different shapes, for instance oblong protein structures (such as the chloride channel in Fig. 3h), ‘winged’ structures (such as ion channels with voltage sensor domains in Fig. 3b), and multimeric proteins.

We explored alternate parameterizations of our algorithm to evaluate our default choices for these parameters. The results in Table 3 demonstrate that the classification of lipid accessibility by our algorithm is quite stable with varying parameter sets. The default parameter set we used for benchmarking was a slice width of 10 Å, a distance cutoff of 10 Å, a shell radius of 6 Å and an angle cutoff of 65°. In addition to the default parameter set, we tested additional values for each parameter. Even with different parameter sets, our algorithm consistently achieves accuracies, sensitivities and specificities around 90%, with two exceptions. If the distance cutoff (i.e. ‘resolution’ of the concave hull) is increased to 15 Å or greater, the algorithm under-predicts lipid accessibility: the number of true positives decreases while the number of false negatives increases. A similar situation occurs when the shell radius is decreased to 4 Å, as fewer residues are classified as lipid exposed.

**Table 3:** Prediction accuracies for different parameter sets, showing the stability of the algorithm

	Slice width	Dist cutoff	Shell radius	Angle cutoff	acc	sens	spec
*Default*	*10*	*10*	*6*	*65*	*91.2*	*89.4*	*91.7*
	*5*	10	6	65	89.5	91.4	88.9
	*7.5*	10	6	65	90.5	90.3	90.6
	10	*5*	6	65	87.4	92.3	86.0
	10	*15*	6	65	91.5	**81.3**	94.5
	10	10	*4*	65	91.9	**82.0**	94.8
	10	10	*8*	65	90.0	91.1	89.7
	10	10	6	*50*	90.9	89.5	91.4
	10	10	6	*80*	91.1	87.8	92.1

*acc* accuracy = (TP + TN)/(TP + TN + FP + FN); sens = TP/(TP + FN); spec = TN/(TN + FP) with *TP* number of residues predicted as true positives, *TN* true negatives, *FP* false positives, *FN* false negatives

The meaning of the parameters is explained in the Methods section. Our algorithm is stable with respect to smaller slice widths (Fig. 1a) and different angle cutoffs (Fig. 1b). For larger distance cutoffs (Fig. 2d and e) and smaller shell radii (from Fig. 2e to f) the number of predicted true positives decreases while the number of false negatives increases, resulting in a substantial drop in sensitivity (numbers in bold)

**Fig. 4:**
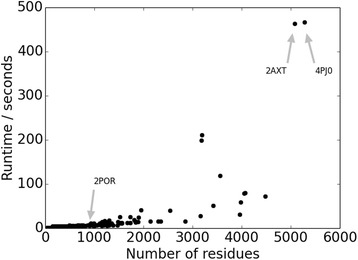
Runtimes of mp_lipid_acc in seconds for proteins of varying sizes. Runtimes are obtained when using the default parameters of a slice width of 10 Å, a distance cutoff of 10 Å, and a shell radius of 6 Å

Runtimes for mp_lipid_acc are consistently short, typically under 1 min for proteins up to 2000 residues, using default parameters (Fig. 4). The algorithm only takes into account Cα atoms in the protein. We tested using all atoms for the calculation of the hulls and the concave shell, which increased the runtimes considerably (over 1 h), especially for large proteins, while achieving similar results. We therefore only make the option available to use Cα atoms.

While improvements to the algorithm might lead to a smaller number of false positives, our algorithm is the first to classify lipid exposed residues from the protein structure, yielding prediction accuracies around 90%. It will be useful for molecular modeling and developing score functions and more sophisticated sequence-based approaches for the prediction of lipid accessibility. Classification of lipid accessibility from the structure is also useful to advance our understanding of membrane protein folding, stability and their interactions in the membrane bilayer [4].

## Conclusion

Here we present a novel method to classify lipid accessibility in membrane protein structures. Our algorithm is applicable to α-helical and β-barrel membrane proteins with and without pores and for diverse protein architectures. mp_lipid_acc is implemented in RosettaMP and uses membrane embedded structures (from PDBTM or OPM) and a fixed membrane thickness to classify residues based on a concave hull algorithm. To test our method, we manually curated a benchmark dataset, on which mp_lipid_acc achieves accuracies, specificities and sensitivities around 90%. We believe that mp_lipid_acc and our benchmark set are an important first step for sequence- and structure-based prediction of lipid accessibility, score function optimization and membrane protein modeling in general.

## Additional file

Additional file 1: Table S1. Dataset for developing the algorithm: The table contains details about each protein in the training dataset. **Table S2.** Test database: The table contains accuracies for each protein in the benchmark dataset. (PDF 453 kb)

## Abbreviations

ABC: ATP-binding cassette
EM: Electron microscopy
GPCR: G-protein coupled receptor
PDB: Protein Data Bank
rASA: Relative accessible surface area
SASA: Solvent-accessible surface area
TM: Trans-membrane
XFEL: X-ray free electron laser

## References

[1] Yildirim MA, Goh K-I, Cusick ME, Barabási A-L, Vidal M (2007). Drug-target network. Nat Biotechnol.

[2] Koehler Leman J, Ulmschneider MB, Gray JJ (2015). Computational modeling of membrane proteins. Proteins Struct Funct Bioinf.

[3] Nguyen D, Helms V, Lee P-H (2014). PRIMSIPLR: Prediction of inner-membrane situated pore-lining residues for alpha-helical transmembrane proteins. Proteins Struct Funct Bioinf.

[4] Hong H (2014). Toward understanding driving forces in membrane protein folding. Arch Biochem Biophys.

[5] Li E, Wimley WC, Hristova K (2012). Transmembrane helix dimerization: Beyond the search for sequence motifs. Biochim Biophys Acta - Biomembr.

[6] van Meer G, Voelker DR, Feigenson GW (2008). Membrane lipids: where they are and how they behave. Nat Rev Mol Cell Biol.

[7] Slusky JSG, Dunbrack RL (2013). Charge asymmetry in the proteins of the outer membrane. Bioinformatics.

[8] Zhou H-X, Cross TA (2013). Influences of membrane mimetic environments on membrane protein structures. Annu Rev Biophys.

[9] Botelho AV, Huber T, Sakmar TP, Brown MF (2006). Curvature and hydrophobic forces drive oligomerization and modulate activity of Rhodopsin in membranes. Biophys J.

[10] Tusnády GE, Dosztányi Z, Simon I (2005). TMDET: web server for detecting transmembrane regions of proteins by using their 3D coordinates. Bioinformatics.

[11] Lomize MA, Pogozheva ID, Joo H, Mosberg HI, Lomize AL (2012). OPM database and PPM web server: resources for positioning of proteins in membranes. Nucleic Acids Res.

[12] Kelm S, Shi J, Deane CM (2009). iMembrane: Homology-based membrane-insertion of proteins. Bioinformatics.

[13] Koehler J, Mueller R, Meiler J (2009). “Improved prediction of trans-membrane spans in proteins using an artificial neural network,” in 2009 IEEE Symposium on Computational Intelligence in Bioinformatics and Computational Biology.

[14] Leman JK, Mueller R, Karakas M, Woetzel N, Meiler J (2013). Simultaneous prediction of protein secondary structure and transmembrane spans. Proteins.

[15] Viklund H, Elofsson A (2008). OCTOPUS: improving topology prediction by two-track ANN-based preference scores and an extended topological grammar. Bioinformatics.

[16] Hayat S, Elofsson A (2012). BOCTOPUS: Improved topology prediction of transmembrane beta-barrel proteins. Bioinformatics.

[17] Yuan Z, Zhang F, Davis MJ, Bodén M, Teasdale RD (2006). Predicting the solvent accessibility of transmembrane residues from protein sequence. J Proteome Res.

[18] Illergård K, Callegari S, Elofsson A (2010). MPRAP: an accessibility predictor for a-helical transmembrane proteins that performs well inside and outside the membrane. BMC Bioinf.

[19] Lai J-S, Cheng C-W, Lo A, Sung T-Y, Hsu W-L (2013). Lipid exposure prediction enhances the inference of rotational angles of transmembrane helices. BMC Bioinf.

[20] Adamian L, Liang J (2006). Prediction of transmembrane helix orientation in polytopic membrane proteins. BMC Struct Biol.

[21] Hubbard SJ, Thornton JM (1993). “Naccess, computer program.” department of biochemistry and molecular biology, university college London.

[22] Sanner MF, Olson AJ, Spehner JC (1996). Reduced surface: an efficient way to compute molecular surfaces. Biopolymers.

[23] Fraczkiewicz R, Braun W (1998). Exact and efficient analytical calculation of the accessible surface areas and their gradients for macromolecules. J Comput Chem.

[24] Voss NR, Gerstein M (2010). 3V: cavity, channel and cleft volume calculator and extractor. Nucleic Acids Res.

[25] Lyskov S, Chou FC, Conchúir SÓ, Der BS, Drew K, Kuroda D, Xu J, Weitzner BD, Renfrew PD, Sripakdeevong P, Borgo B, Havranek JJ, Kuhlman B, Kortemme T, Bonneau R, Gray JJ, Das R (2013). Serverification of molecular modeling applications: the Rosetta Online Server that Includes Everyone (ROSIE). PLoS One.

[26] Kozma D, Simon I, Tusnády GE (2013). PDBTM: Protein Data Bank of transmembrane proteins after 8 years. Nucleic Acids Res.

[27] Wang G, Dunbrack RL (2005). PISCES : recent improvements to a PDB sequence culling server. Nucleic Acids Res.

[28] Tusnády GE, Dosztányi Z, Simon I (2004). Transmembrane proteins in the Protein Data Bank: identification and classification. Bioinformatics.

[29] Alford RF, Koehler Leman J, Weitzner BD, Duran AM, Tilley DC, Elazar A, Gray JJ (2015). An integrated framework advancing membrane protein modeling and design. PLoS Comput Biol.

[30] The PyMOL Molecular Graphics System, Version 1.8 Schrödinger, LLC. http://pymol.org/citing.

[31] de Berg M, Cheong O, van Kreveld M, and Overmars M, Computational Geometry - Algorithms and Applications, Third Edit. Berlin: Springer; 2008.

[32] Barber CB, Dobkin DP, Huhdanpaa H (1996). The quickhull algorithm for convex hulls. ACM Trans Math Softw.

[33] Rosen E, Jansson E, Brundin M (2014). “Implementation of a fast and efficient concave hull algorithm,” Uppsala Univ.

[34] Kabsch W, Sander C (1983). Dictionary of protein secondary structure: pattern recognition of hydrogen-bonded and geometrical features. Biopolymers.

