# Supplementary material for: Computing structure-based lipid accessibility of membrane proteins with mp_lipid_acc in RosettaMP
Source: BMC Bioinformatics. 2017 Feb 20;18:115. doi: 10.1186/s12859-017-1541-z (PMC5319049; doi:10.1186/s12859-017-1541-z)
Supplement: Additional file 1: Table S1. — Dataset for developing the algorithm: The table contains details about each protein in the training dataset. Table S2. Test database: The table contains accuracies for each protein in the benchmark dataset. (PDF 453 kb) [file 12859_2017_1541_MOESM1_ESM.pdf]

Additional file 1 for:

Computing structure-based lipid accessibility of membrane proteins with *mp_lipid_acc* in RosettaMP

Julia Koehler Leman[1,2]*, Sergey Lyskov[3], Richard Bonneau[1,2]

[1] Center for Computational Biology, Flatiron Institute, Simons Foundation, 162 Fifth Avenue, New York, NY 10010, USA
[2] Departments of Biology and Computer Science, Center for Genomics and Systems Biology, New York University, New York, NY 10003, USA
[3] Department of Chemical and Biomolecular Engineering, Johns Hopkins University, Baltimore, MD 21218, USA
* corresponding author: julia.koehler.leman@gmail.com

Usage:

RosettaMP's `mp_lipid_acc` application requires the protein to be transformed into membrane coordinates. This can be done by downloading the PDB from the PDBTM [1] or the OPM database [2]. The PDB then needs to be cleaned and renumbered, and the Rosetta span file needs to be computed. Details of these steps can be found in the supplement of reference [3].

The detailed command line of the `mp_lipid_acc` application is:

```
# executable
Rosetta/main/source/bin/mp_lipid_acc.macosclangrelease \

# Rosetta database
-database Rosetta/main/database \

# required: PDB input file
-in:file:s 5IRX__tr.pdb \

# required: Rosetta span file
-mp:setup:spanfiles 5IRX__tr.span \

# optional: defines the width of the horizontal slices in Angstrom; default 10 Å
# the membrane thickness should be an integer multiple of this number
-mp:lipid_acc:slice_width 10 \

# optional: distance cutoff to define the 'resolution' of the concave hull; default 10 Å
-mp:lipid_acc:dist_cutoff 10 \

# optional: shell radius that defines the thickness of the shell of residues that should be classified as
# lipid accessible with respect to the points of the concave hull; default 6 Å
-mp:lipid_acc:shell_radius 6 \

# optional: is the protein's lipid exposed area helical or not? The type is auto-detected, but can be overwritten
# with this option
-mp:lipid_acc:tm_alpha false \
```

# optional expert flag for β-barrels (and helical bundles with less or equal 7 TM spans): residues with angles
# between Cβ-Cα-COM that are larger than the cutoff are classified as lipid exposed
# default for β-barrels: 65 degrees; default for small α-helical bundles: 45 degrees

```
-mp:lipid_acc:angle_cutoff 65 \
```

The application creates a PDB file with an adjusted B-factor column. The output PDB can then be visualized in PyMOL with the provided script `color_b-factor.pml`: open the PDB in PyMOL and then run

```
@color_b-factor.pml
```

in the viewer or command line window inside PyMOL.

Table S1: Dataset for developing the algorithm

PDBID	protein	A/B	#subunits	#TM total	#residues
1AFO	glycophorin A	A	2	2	80
2KIX	M2 channel	A	4	4	132
1RH1	β2-adrenergic receptor	A	1	7	442
3WMF	ABC transporter	A	2	12	1176
4TNW	glutamate-gated chloride channel	A	5	20	1703
1KPK	chloride channel	A	2	28	900
2R9R	voltage-gated potassium channel	A	4	28	1544
3NE2	aquaporin	A	4	32	978
2WCD	cytolysin A	A	12	36	3420
1EK9	TolC	B	3	12	1284
7AHL	α-hemolysin	B	7	14	2051
3EMN	VDAC	B	1	19	283
1FEP	outer membrane receptor FepA	B	1	22	680
1QD6	outer membrane phospholipase A	B	2	24	479

Table S2: Test database

Proteins, their secondary structure type (A/B) and predictions with `mp_lipid_acc` on the benchmark dataset, compared to manually curated lipid accessibilities. Columns 3-6 are the number of residues that are true positives, true negatives, false positives and false negatives. The last three columns are prediction accuracies, sensitivities, and specificities in %.

protein	A/B	TP	TN	FP	FN	acc	sens	spec
1C17	A	260	669	112	48	85.3	84.4	85.7
1FDM	A	33	16	0	0	100.0	100.0	100.0
1H2S	A	244	199	112	12	78.1	95.3	64.0
1KF6	A	224	1835	57	21	96.4	91.4	97.0
1KMO	B	138	503	12	7	97.1	95.2	97.7
1M0K	A	207	311	126	21	77.9	90.8	71.2
1OH2	B	244	931	46	17	94.9	93.5	95.3
1P49	A	44	503	0	0	100.0	100.0	100.0
1PPJ	A	433	3394	88	65	96.2	86.9	97.5
1QJP	B	54	75	6	1	94.9	98.2	92.6
1RZH	A	233	511	39	36	90.8	86.6	92.9
1U19	A	98	212	27	10	89.3	90.7	88.7
1U7G	A	275	697	124	52	84.7	84.1	84.9
1UUN	B	120	1295	0	56	96.2	68.2	100.0
1UYN	B	73	202	0	3	98.9	96.1	100.0
1XKW	B	140	501	9	4	98.0	97.2	98.2
2A65	A	281	556	148	34	82.1	89.2	79.0
2BL2	A	280	969	100	210	80.1	57.1	90.6
2BS2	A	171	2047	53	24	96.6	87.7	97.5
2CFQ	A	147	178	74	8	79.9	94.8	70.6
2F2B	A	251	584	121	23	85.3	91.6	82.8
2FGQ	B	209	742	31	7	96.2	96.8	96.0
2GR8	B	66	151	14	2	93.1	97.1	91.5
2GUF	B	139	400	4	7	98.0	95.2	99.0
2J58	A	125	2637	71	14	97.0	89.9	97.4
2JLN	A	146	246	52	18	84.8	89.0	82.6
2K73	A	81	78	10	13	87.4	86.2	88.6
2KLU	A	21	46	0	2	97.1	91.3	100.0
2KNC	A	22	31	0	0	100.0	100.0	100.0
2KOG	A	21	94	0	0	100.0	100.0	100.0
2KS9	A	101	213	30	18	86.7	84.9	87.7
2KSD	A	50	24	0	0	100.0	100.0	100.0
2KSE	A	46	27	0	3	96.1	93.9	100.0
2KSF	A	64	28	7	7	86.8	90.1	80.0
2L0J	A	63	71	22	7	82.2	90.0	76.3
2L2T	A	44	41	0	2	97.7	95.7	100.0
2L35	A	46	39	8	1	90.4	97.9	83.0
2LCK	A	101	162	27	6	88.9	94.4	85.7
2LHF	B	65	102	7	3	94.4	95.6	93.6

2LZL	A	22	20	0	0	100.0	100.0	100.0
2M67	A	39	41	0	0	100.0	100.0	100.0
2MFR	A	21	35	0	0	100.0	100.0	100.0
2MIC	A	53	28	0	0	100.0	100.0	100.0
2MMU	A	42	6	0	1	98.0	97.7	100.0
2MOF	A	19	22	0	0	100.0	100.0	100.0
2MPN	A	70	48	10	7	87.4	90.9	82.8
2MPR	B	238	966	49	9	95.4	96.4	95.2
2MXB	A	20	27	0	1	97.9	95.2	100.0
2N2A	A	42	73	0	0	100.0	100.0	100.0
2N4X	A	105	50	34	2	81.2	98.1	59.5
2NQ2	A	230	762	95	19	89.7	92.4	88.9
2NWL	A	352	611	207	35	79.9	91.0	74.7
2POR	B	224	621	37	20	93.7	91.8	94.4
2QKS	A	149	974	63	21	93.0	87.6	93.9
2QTS	A	97	1126	17	14	97.5	87.4	98.5
2VDF	B	62	155	3	4	96.9	93.9	98.1
2VPZ	A	225	2009	106	13	94.9	94.5	95.0
2WDQ	A	253	2789	85	28	96.4	90.0	97.0
2WJR	B	76	120	4	3	96.6	96.2	96.8
2WSW	A	451	832	163	77	84.2	85.4	83.6
2X27	B	49	148	4	7	94.7	87.5	97.4
2X55	B	68	202	1	2	98.9	97.1	99.5
2X9K	B	91	171	4	11	94.6	89.2	97.7
2XFN	A	51	447	0	0	100.0	100.0	100.0
2XOV	A	94	49	23	14	79.4	87.0	68.1
2YEV	A	244	741	149	28	84.8	89.7	83.3
2YNK	B	121	299	8	12	95.5	91.0	97.4
2ZFG	B	229	728	50	9	94.2	96.2	93.6
2ZXE	A	187	1041	52	15	94.8	92.6	95.2
3AG3	A	528	2653	333	43	89.4	92.5	88.8
3AR4	A	158	762	57	16	92.6	90.8	93.0
3B9W	A	250	668	137	30	84.6	89.3	83.0
3BS0	B	83	309	10	11	94.9	88.3	96.9
3CSL	B	138	769	7	1	99.1	99.3	99.1
3CX5	A	413	3886	90	88	96.0	82.4	97.7
3D31	A	145	947	86	13	91.7	91.8	91.7
3DH4	A	308	493	138	50	81.0	86.0	78.1
3DWO	B	79	343	12	9	95.3	89.8	96.6
3DZM	B	53	150	2	2	98.1	96.4	98.7
3EGW	A	217	3644	55	39	97.6	84.8	98.5
3FHH	B	141	469	6	4	98.4	97.2	98.7
3FID	B	80	196	4	15	93.6	84.2	98.0
3GIA	A	158	200	65	9	82.9	94.6	75.5
3GP6	B	64	75	10	5	90.3	92.8	88.2
3K3F	A	290	550	91	64	84.4	81.9	85.8

3KCU	A	316	754	75	94	86.4	77.1	91.0
3KVN	B	72	551	0	4	99.4	94.7	100.0
3LDC	A	170	71	58	28	73.7	85.9	55.0
3M73	A	275	463	154	40	79.2	87.3	75.0
3NE5	A	435	3366	168	80	93.9	84.5	95.2
3O44	B	210	3781	0	75	98.2	73.7	100.0
3PCV	A	147	209	75	6	81.5	96.1	73.6
3QRA	B	47	98	2	4	96.0	92.2	98.0
3RFZ	B	151	1084	11	10	98.3	93.8	99.0
3RLF	A	202	1598	64	26	95.2	88.6	96.1
3RQW	A	224	1224	66	20	94.4	91.8	94.9
3S8G	A	174	467	87	22	85.5	88.8	84.3
3SZV	B	126	221	11	5	95.6	96.2	95.3
3TUI	A	137	897	69	15	92.5	90.1	92.9
3UG9	A	188	273	94	4	82.5	97.9	74.4
3V8X	B	131	1382	6	9	99.0	93.6	99.6
3VW7	A	96	299	28	18	89.6	84.2	91.4
3VY8	B	227	747	28	20	95.3	91.9	96.4
3W4T	A	155	196	81	13	78.9	92.3	70.8
3WAJ	A	168	551	61	12	90.8	93.3	90.0
3WFD	A	165	343	67	15	86.1	91.7	83.7
3WXW	A	100	141	28	13	85.5	88.5	83.4
3X2R	B	165	1550	9	6	99.1	96.5	99.4
3ZE3	A	124	123	45	11	81.5	91.9	73.2
3ZOJ	A	283	639	89	36	88.1	88.7	87.8
4A01	A	266	1018	158	37	86.8	87.8	86.6
4A82	A	160	903	86	6	92.0	96.4	91.3
4AFK	B	110	306	7	6	97.0	94.8	97.8
4AL0	A	156	203	51	27	82.2	85.2	79.9
4B7O	B	138	498	10	2	98.1	98.6	98.0
4C69	B	120	148	7	7	95.0	94.5	95.5
4COF	A	226	1304	123	11	91.9	95.4	91.4
4D5B	B	81	224	0	6	98.1	93.1	100.0
4DVE	A	94	60	21	13	81.9	87.9	74.1
4DX5	A	346	2805	231	35	92.2	90.8	92.4
4E1S	B	77	157	4	3	97.1	96.3	97.5
4EIY	A	98	251	28	12	89.7	89.1	90.0
4ENE	A	257	485	115	28	83.8	90.2	80.8
4FQE	B	75	89	0	5	97.0	93.8	100.0
4G7V	A	74	42	9	10	85.9	88.1	82.4
4GEY	B	114	286	8	11	95.5	91.2	97.3
4GX0	A	160	1639	32	12	97.6	93.0	98.1
4HFI	A	242	1218	81	13	94.0	94.9	93.8
4I0U	A	158	1515	33	23	96.8	87.3	97.9
4IKV	A	174	218	84	15	79.8	92.1	72.2
4J05	A	145	193	73	10	80.3	93.5	72.6

4K1C	A	157	142	58	16	80.2	90.8	71.0
4KNF	A	430	474	210	31	79.0	93.3	69.3
4KPP	A	167	138	68	21	77.4	88.8	67.0
4KYT	A	149	791	56	7	93.7	95.5	93.4
4MEE	B	72	225	0	5	98.3	93.5	100.0
4MES	A	90	52	22	11	81.1	89.1	70.3
4MSW	A	181	183	31	12	89.4	93.8	85.5
4N75	B	115	258	1	4	98.7	96.6	99.6
4N7W	A	260	223	93	34	79.2	88.4	70.6
4NV5	A	86	144	14	14	89.1	86.0	91.1
4O6M	A	168	404	82	16	85.4	91.3	83.1
4O6Y	A	167	166	66	21	79.3	88.8	71.6
4O93	A	294	256	125	32	77.8	90.2	67.2
4OGQ	A	358	1326	110	103	88.8	77.7	92.3
4P02	A	190	1116	51	25	94.5	88.4	95.6
4P79	A	65	98	12	5	90.6	92.9	89.1
4PD6	A	386	616	135	65	83.4	85.6	82.0
4PGR	A	104	66	22	14	82.5	88.1	75.0
4PHZ	A	415	1912	183	24	91.8	94.5	91.3
4PL0	A	154	909	66	17	92.8	90.1	93.2
4Q35	B	167	700	16	24	95.6	87.4	97.8
4QL0	B	107	400	1	11	97.7	90.7	99.8
4QND	A	84	65	29	15	77.2	84.8	69.1
4QTN	A	255	319	111	21	81.3	92.4	74.2
4QUV	A	171	157	40	24	83.7	87.7	79.7
4R0C	A	274	505	102	62	82.6	81.5	83.2
4RDQ	A	221	1505	79	24	94.4	90.2	95.0
4RDR	B	137	557	5	6	98.4	95.8	99.1
4RI2	A	156	134	24	25	85.5	86.2	84.8
4RJW	B	229	971	61	16	94.0	93.5	94.1
4RL8	B	80	179	2	5	97.4	94.1	98.9
4RLC	B	52	75	3	4	94.8	92.9	96.2
4RP9	A	303	436	121	41	82.0	88.1	78.3
4RYO	A	75	51	16	8	84.0	90.4	76.1
4TQ3	A	131	86	61	4	77.0	97.0	58.5
4TWK	A	179	253	42	21	87.3	89.5	85.8
4U4V	A	145	221	64	11	83.0	92.9	77.5
4U9N	A	129	141	67	17	76.3	88.4	67.8
4UC1	A	129	110	54	19	76.6	87.2	67.1
4UMW	A	125	434	32	11	92.9	91.9	93.1
4UVM	A	194	203	87	20	78.8	90.7	70.0
4V1G	A	153	560	36	18	93.0	89.5	94.0
4WD8	A	191	1061	82	10	93.2	95.0	92.8
4WW3	A	100	199	32	18	85.7	84.7	86.1
4X5M	A	87	66	19	13	82.7	87.0	77.6
4X89	A	183	123	49	8	84.3	95.8	71.5

4XES	A	101	321	33	15	89.8	87.1	90.7
4XNV	A	94	194	29	22	85.0	81.0	87.0
4XP9	A	161	296	65	14	85.3	92.0	82.0
4XTL	A	96	128	35	10	83.3	90.6	78.5
4XU4	A	252	192	108	14	78.4	94.7	64.0
4Y25	B	100	179	3	7	96.5	93.5	98.4
4ZP0	A	142	156	71	22	76.2	86.6	68.7
4ZR1	A	83	160	9	21	89.0	79.8	94.7
4ZW9	A	146	234	72	17	81.0	89.6	76.5
5A1S	A	276	440	104	38	83.4	87.9	80.9
5AJI	A	366	1325	71	46	93.5	88.8	94.9
5AWW	A	193	268	65	32	82.6	85.8	80.5
5AYN	A	154	159	78	13	77.5	92.2	67.1
5BZ3	A	280	344	104	39	81.4	87.8	76.8
5CKR	A	208	304	106	39	77.9	84.2	74.1
5DQQ	A	431	696	107	63	86.9	87.2	86.7
5EKE	A	133	995	40	21	94.9	86.4	96.1
5EZM	A	194	260	60	22	84.7	89.8	81.3
5FOK	B	135	504	5	11	97.6	92.5	99.0
5FXB	A	125	241	47	8	86.9	94.0	83.7
5HK1	A	33	172	0	6	97.2	84.6	100.0
5HYA	A	149	86	48	13	79.4	92.0	64.2
5I20	A	145	69	60	12	74.8	92.4	53.5
5IRX	A	391	1139	143	52	88.7	88.3	88.8
5IVA	B	161	551	12	6	97.5	96.4	97.9
5IWS	A	263	464	119	39	82.1	87.1	79.6
5IXM	B	166	496	10	11	96.9	93.8	98.0
all		33191	116548	10585	3937	91.2	89.4	91.7

References:

[1] D. Kozma, I. Simon, and G. E. Tusnady, “PDBTM: Protein Data Bank of transmembrane proteins after 8 years,” *Nucleic Acids Res.*, vol. 41, no. D1, pp. D524–D529, 2013.

[2] M. A. Lomize, I. D. Pogozheva, H. Joo, H. I. Mosberg, and A. L. Lomize, “OPM database and PPM web server: resources for positioning of proteins in membranes.,” *Nucleic Acids Res.*, vol. 40, no. Database issue, pp. D370-6, Jan. 2012.

[3] R. F. Alford, J. Koehler Leman, B. D. Weitzner, A. M. Duran, D. C. Tilley, A. Elazar, and J. J. Gray, “An Integrated Framework Advancing Membrane Protein Modeling and Design.,” *PLoS Comput. Biol.*, vol. 11, no. 9, p. e1004398, Sep. 2015.
